# Gold Nanorods-Based Photothermal Therapy: Interactions Between Biostructure, Nanomaterial, and Near-Infrared Irradiation

**DOI:** 10.1186/s11671-022-03706-3

**Published:** 2022-07-26

**Authors:** Ruili Zhou, Meigui Zhang, Jiahui Xi, Jing Li, Ruixia Ma, Longfei Ren, Zhongtian Bai, Kuo Qi, Xun Li

**Affiliations:** 1 The First School of Clinical Medicine, Lanzhou University, No. 1 Donggang West Road, Lanzhou, 730000 Gansu Province China; 2 Key Laboratory of Biotherapy and Regenerative Medicine of Gansu Province, Lanzhou, 730000 China; 3 Department of General Surgery, The First Hospital of Lanzhou University, Lanzhou, 730000 China; 4 Hepatopancreatobiliary Surgery Institute of Gansu Province, Medical College Cancer Center of Lanzhou University, Lanzhou, 730000 China

**Keywords:** Photothermal therapy, Gold nanorods, Tumors, Nano–bio interactions, Functionalization, Near-infrared irradiation

## Abstract

Gold nanorods (AuNRs) are ideal inorganic nanophotothermal agents with unique characteristics, including local surface plasmon resonance effects, easy scale preparation and functional modification, and good biocompatibility. This review summarizes several recent advances in AuNRs-based photothermal therapy (PTT) research. Functionalized AuNRs photothermal agents have optimized biocompatibility and targeting properties. The multifunctional AuNRs nanoplatform composite structure meets the requirements for synergistic effects of PTT, photoacoustic imaging, and other therapeutic methods. Photothermal therapy with AuNRs (AuNRs-PTT) is widely used to treat tumors and inflammatory diseases; its tumor-targeting, tumor metastasis inhibition, and photothermal tumor ablation abilities have remarkable curative effects. An in-depth study of AuNRs in living systems and the interactions between biological structure, nanomaterial, and near-infrared irradiation could lay the foundation for further clinical research and the broad application of AuNRs in PTT.

## Introduction

**Fig. 1:**
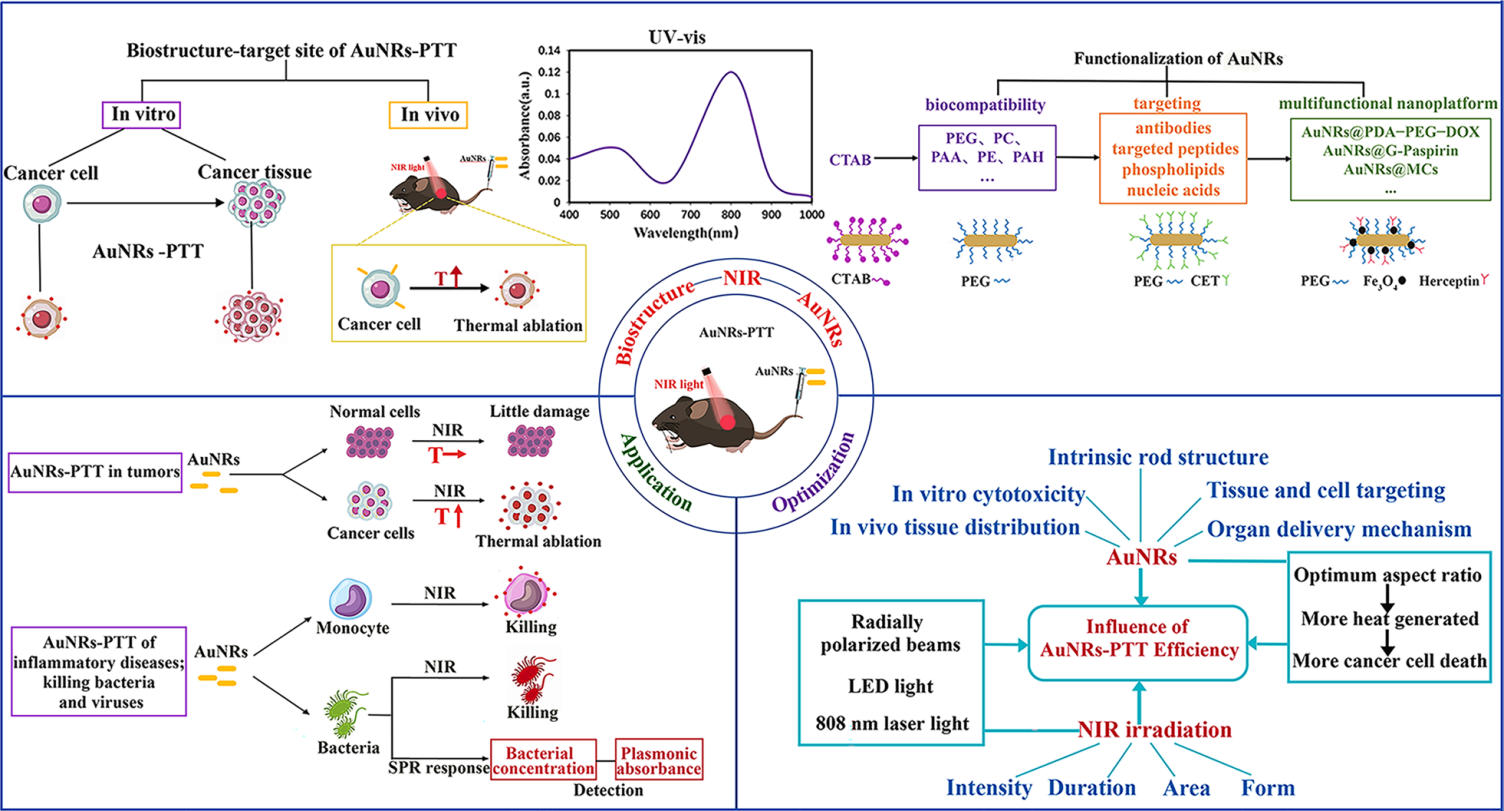
Gold nanorods-based photothermal therapy: interactions between biostructure, nanomaterial, and near-infrared irradiation

Scientists are no longer satisfied with current surgical treatment, radiation, chemo, biological, immuno, gene, and targeted molecular therapies. They are currently focusing on applying advanced nanotechnology to clinical diagnosis and treatment, exploring the physical advantages of medical–biological nanomaterials [1]. Nanophotothermal therapy organically combines biological structure, nanomaterial, and NIR irradiation based on traditional hyperthermia to utilize the photothermal conversion performance of nanophotothermal agents under the action of external light irradiation to cure diseases noninvasively [2]. AuNRs are ideal inorganic nanophotothermal agents whose characteristics include high photothermal conversion efficiency, good biocompatibility, easy surface functionalization, simple and controllable preparation schemes, and adjustable NIR absorption spectra, so AuNRs occupy an important position in nanophotothermal therapy research [3, 4]. To date, AuNRs-PTT research has covered a broad scope, including the structure and functional properties of the material; in vitro and in vivo experiments on photothermal agents; its effect on malignant tumors, bacteria, and viruses; cell and molecular biology; and multidisciplinary interdisciplinary studies [5]. This review focuses on the structural and functional characteristics of AuNRs as nanophotothermal agents and their application in nanophotothermal tumor diagnosis and treatment, discusses the interaction between nanostructures and cells, as well as the optimization of the intrinsic structure of AuNRs and the improvement in external field irradiation conditions, and summarizes and prospects for AuNRs photothermal-agent-based PTT research (Fig. 1).

## Nanophotothermal Therapy

### Hyperthermia Therapy and Thermal Ablation

Thermal medicine involves manipulating body or tissue temperatures to treat diseases. The therapeutic heating of tumor tissue can be divided into hyperthermia and thermal ablation [6]. Hyperthermia therapy induces the heating of relatively light tumors to a maximum of 45 °C, while thermal ablation generates temperatures exceeding 50 °C, lasting several minutes during a single treatment session, destroying tumor cells by heat alone [7]. Based on clinical studies, hyperthermia and thermal ablation therapies have been shown to have many tumor indications in clinical oncology; the former is used for advanced local cervical cancer, non-muscle-invasive bladder cancer, recurrent breast cancer, and soft tissue sarcoma that are not indicated for chemotherapy; the clinical indications of the latter include hepatocellular carcinoma, primary and secondary lung cancer, prostate cancer, renal cell carcinoma (the most common kidney cancer), and non-surgical liver and adrenal metastases [8–10].

### PTT, Nanophotothermal Agents, and NIR Irradiation

PTT uses a laser beam to heat contrast agents (photothermal conversion nanomaterials) introduced into tissues and cells to cause thermal damage in areas of interest, such as tumor tissue. One of the keys to nanophotothermal therapy is nanophotothermal agent selection [11]. Nanomaterials in the 20–300 nm range can preferentially aggregate in tumor tissues due to their enhanced permeability and retention (EPR) effect [12]. Widely used nanophotothermal agents include noble metals (primarily gold), semiconductors (including quantum dot and two-dimensional materials), carbon-based, and organic polymer nanomaterials [13, 14]. The photothermal conversion efficiency is an important indicator for the selection of an ideal photothermal agent. For gold nanocrystals, the photothermal conversion efficiency strongly depends on the plasmonic resonance wavelength, nanostructure volume, shell coating, and assembly state [15]. The research of directly measuring the temperature of the gold nanocrystal solution by thermocouple found that when the plasmon resonance wavelength is the same as the wavelength of the irradiated laser, a high photothermal conversion efficiency is produced, and the photothermal conversion efficiency is inversely proportional to the volume of nanocrystal particles with the same shape [15]. The surface coating of nanocrystals with appropriate materials can improve the plasmonic photothermal conversion efficiency, and the assembly of gold nanocrystals is an effective means to control its photothermal conversion performance [15]. In practical applications, the biological environment of the nanophotothermal agent will affect the maintenance of the crystal structure, which together with the optical response of the environment itself will jointly affect the photothermal conversion efficiency. NIR light penetrates deeply into the human body with minimal interference from tissue and blood scattering, absorption, and autofluorescence, making it useful in PTT and in vivo PAI. In particular, NIR light located in the transparent biological windows 650 − 950 nm (NIR-I window) and 1000 − 1350 nm (NIR-II window) enables the imaging of deep tissues with a high signal-to-noise ratio [16].

## AuNRs

### Preparation and Characterization of AuNRs

**Fig. 2:**
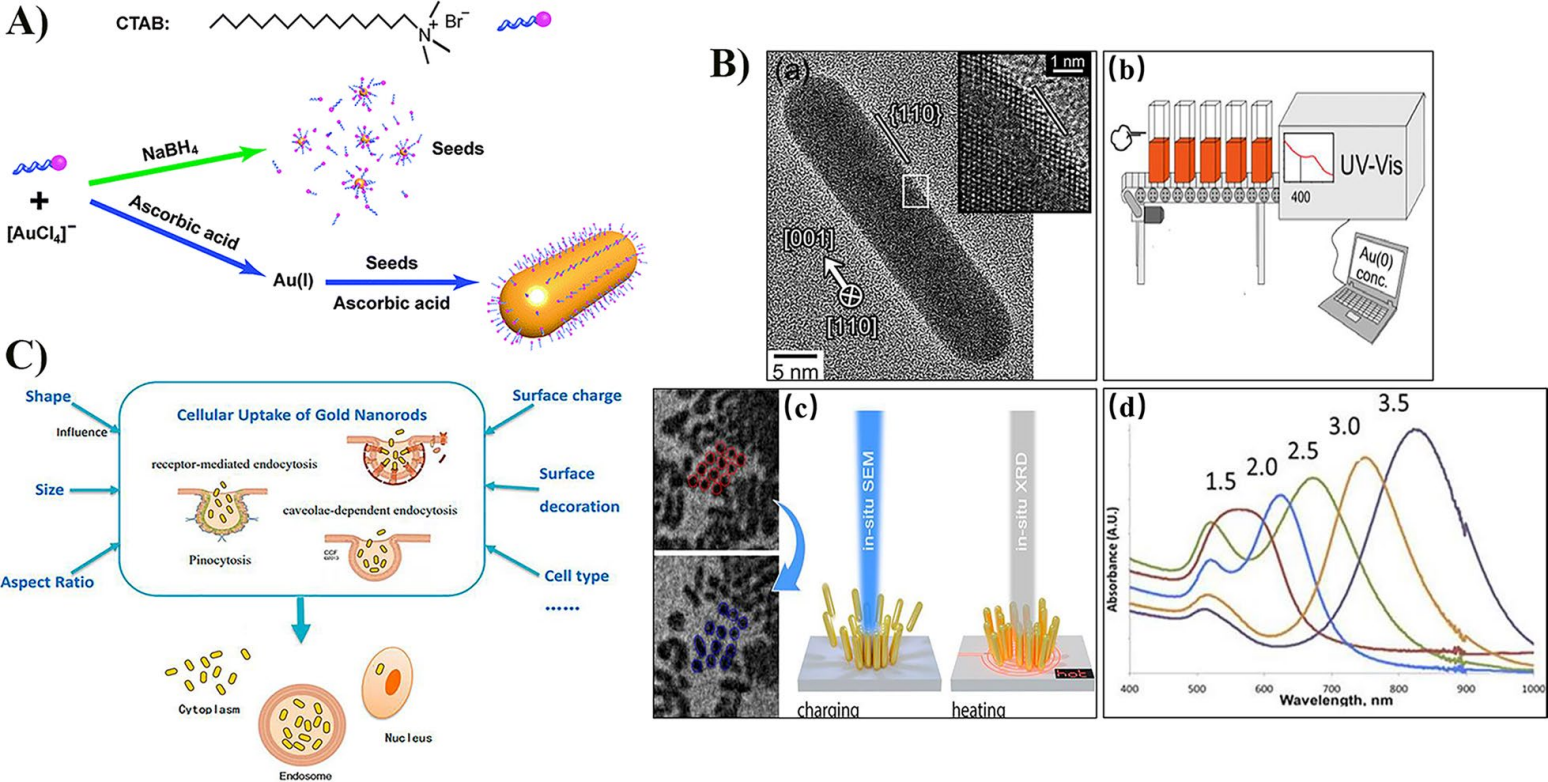
Synthesis, characterization, and uptake of AuNRs. **A** Schematic illustration of the seed-mediated method for the growth of AuNRs. Reproduced with permission from Ref. [17]. Copyright 2013, Royal Society of Chemistry. **B** (a) The AuNRs intrinsic structure was characterized by high-resolution transmission electron microscopy as a single crystal along the [001] long axis. Reproduced with permission from Ref. [23]. Copyright 2019, Oxford University Press. (b) The UV–Vis absorbance of colloidal gold nanoparticles at 400 nm can determine Au concentrations in colloidal gold solutions. Reproduced with permission from Ref. [26]. Copyright 2014, ACS Publication. (c) A picture of the dynamics of vertically aligned assemblies of AuNRs. Reproduced with permission from Ref. [27]. Copyright 2020, ACS Publication. (d) UV–Vis-NIR extinction spectra of AuNRs with aspect ratios (AR) 1.5–3.5, AR are indicated above the absorbance spectrum of each sample. Reproduced with permission from Ref. [5]. Copyright 2021, Frontiers Media S.A. **C** Illustration of several factors affecting the cellular uptake of AuNRs. Reproduced with permission from Ref. [5]. Copyright 2021, Frontiers Media S.A.

AuNRs are mainly prepared using seed growth, template, photochemical, electrochemical, and seedless growth methods [17]. The seed growth method exhibits good controllability and high experimental repeatability and produces high-quality, small-sized AuNRs (Fig. 2A) [18, 19]. The reducing agents commonly used in this method are ascorbic acid and hydroquinone, with cetyltrimethylammonium bromide (CTAB) as a stabilizer during the synthesis to consume gold ions and reducing agents [20]. CTAB toxicity is the main drawback of the seed growth method; therefore, it is particularly important to control the synthesis and structural modulation of AuNRs using low CTAB concentrations [21]. In addition, silver nitrate (AgNO_3_) or hydrogen chloride (HCl) can be used to optimize the nanorod growth conditions by varying the number of seeds [22]. The biocompatibility and structural stability of AuNRs directly determine their photothermal conversion efficiency and potential for applications in medical biology.

High-resolution electron microscopy, atomic force microscopy, and ultraviolet–visible spectroscopy (UV–Vis) are used to characterize the micro–nano-structure, surface state, chemical composition, degree of dispersion, and suspended concentration of AuNRs (Fig. 2B). The structural response of AuNRs to NIR irradiation is the basis of their application in PTT. High-resolution transmission electron microscopy has characterized the intrinsic structure of AuNRs as a single crystal with its long axis along the [001] direction (Fig. 2B-a). Under irradiation with 1064 nm NIR pulsed laser light, the crystal structure changed from rod- to cylindrical-shaped. Time change quantitative analysis showed that pulsed laser-induced atomic excitation and heating of AuNRs were not uniform, indicating that pulsed laser light had a significant effect on the external structure of AuNRs [23]. The Au concentration is crucial for the biological application and related toxicological discussions on AuNRs because it affects AuNRs formation mechanisms, surface modifications, and particle assembly processes [24, 25]. The 400 nm UV–Vis absorbance of colloidal gold nanoparticles can be used to determine the Au concentration of colloidal gold solutions (Fig. 2B-b) [26]. The interaction between AuNRs and the stability of their assembly influences the construction of nanostructure-based multifunctional platforms with unique optical properties at 2D and 3D scales; in situ environmental scanning transmission electron microscopy can reveal single-particle resolution, structural details in bulk samples, and the forces governing the disassembly process (Fig. 2B-c) [27, 28].

### Optical Properties of AuNRs

A major feature of gold nanocrystals is their localized surface plasmon resonance (LSPR) effect [4]. LSPR effect is electromagnetic modes associated with collective oscillations of free electrons confined within the nanoscale [11]. Under resonant excitation, gold nanocrystals possess the unique ability to concentrate the free-space optical field in the subwavelength region near their surface, greatly enhancing the electric field around the nanocrystal, leading to various new mechanisms of light–matter interactions such as plasmon-enhanced spectroscopy, higher harmonic generation, optical nanoantenna effects, and plasmons [17]. Among the numerous gold nanostructures, AuNRs exhibit strong optical extinction properties in the visible and NIR light regions, and their plasmonic absorption bands are divided into two: the longitudinal plasmonic bands, corresponding to light absorption along the long axis of the AuNRs, and the transverse plasmonic bands, corresponding to light absorption along the short axis [29, 30]. When the AuNRs are irradiated with laser light, the phase of coherently excited electrons disappears rapidly in femtoseconds, and then the energy is converted into the phonon reservoir to cause lattice vibrations in about a picosecond. The lattice vibrations conduct energy to the surrounding environment in the order of a hundred picoseconds, thereby heating up the AuNRs [31]. The resonant behavior of AuNRs is affected by its aspect ratio; the stepwise increase in the aspect ratio of AuNRs shifts the longitudinal plasmon absorption peak from the visible region to the NIR region, allowing AuNRs to convert light energy into heat energy under 800–1200 nm NIR laser irradiation that selectively kills tumor cells (Fig. 2B-d) [32]. AuNRs are biocompatible, optically active light absorbers and scatterers, and malignant tumor cells that absorb AuNRs require lower laser energy to induce death than non-malignant cells [30, 33].

### Interaction Between AuNRs and Cells

The interaction between AuNRs and cells determines the cytotoxicity of the nanomaterial and the process of AuNRs uptake by cells; it affects the biosafety of AuNRs and the degradation and clearance pathways in the body [34, 35]. AuNRs synthesis schemes and purification processes can affect the nanorod aspect ratio, CTAB content, surface coating, surface charge, and cell culture medium stability, regulating cellular uptake processes and cell viability, ultimately determining the nanorod–cell interaction (Fig. 2C) [36–39]. In contrast to the previous understanding that nanoparticles are transported through the intercellular space of tumor vascular endothelial cells, recent studies have confirmed that most nanoparticles enter tumors through the active processes of endothelial cells [40]. The localization of AuNRs in the nucleus may also affect the interaction and dynamics of other regulatory molecules, including transcription factors and histones, with genomic DNA [41]. AuNRs enter cells via endocytosis, and their surface modification and the cell type affect the endocytic pathway, with cell type playing a preferential role. The cellular uptake of AuNRs is also influenced by serum proteins and cell surface coating status [42, 43]. In contrast to neutral and negatively charged AuNRs, positively charged AuNRs undergo extensive aggregation when exposed to isolated human skin [44]. Neutral and cationic polyethylene glycol (PEG)-coated AuNRs are superior to anionic and bovine serum albumin (BSA)-coated AuNRs in cytotoxicity, cellular uptake, and wound healing in human dermal fibroblasts [45].

Exploiting the interactions between AuNRs and cells enables the identification of M1 and M2 macrophages in vitro and in vivo, promotes cell proliferation and cancer cell death, and modulates the cellular activity and behavior [46, 47]. The complex system of PEG and terminal amine-functionalized AuNRs can regulate the differentiation of adipose tissue-derived human mesenchymal stem cells into neural-like progenitor cells [48]. On interaction with HepG2 human hepatoma cells, the AuNR-based nucleocapsid structure, Au@Ag NRs, promotes reactive oxygen species (ROS) production. ROS can inhibit the AKT-mTOR signaling pathway and activate autophagy that may have potential adverse health effects on the human body [49, 50]. Photothermal-mediated cell dissociation is a promising method for cell recovery in vitro culture. Cell culture substrates functionalized with AuNRs produce an LSPR-induced nanoscale heating effect when irradiated with biocompatible low-intensity NIR light that triggers shedding adherent mesenchymal stem cells [51].

## Functionalized AuNRs Photothermal Agents

To increase the in vivo photothermal effect, functionalized AuNRs photothermal agents should have good biocompatibility and colloidal stability, no influence on AuNRs optical properties, and longtime in vivo circulation and targeting. Functionalized AuNRs photothermal agents can circulate in vivo and accumulate in the target area, increasing cellular uptake rate, prolonging blood circulation time, increasing biocompatibility, and enhancing nanophotothermal agents targeting and accumulation in the target tissue.

### Optimization of Biocompatibility and Targeting

**Fig. 3:**
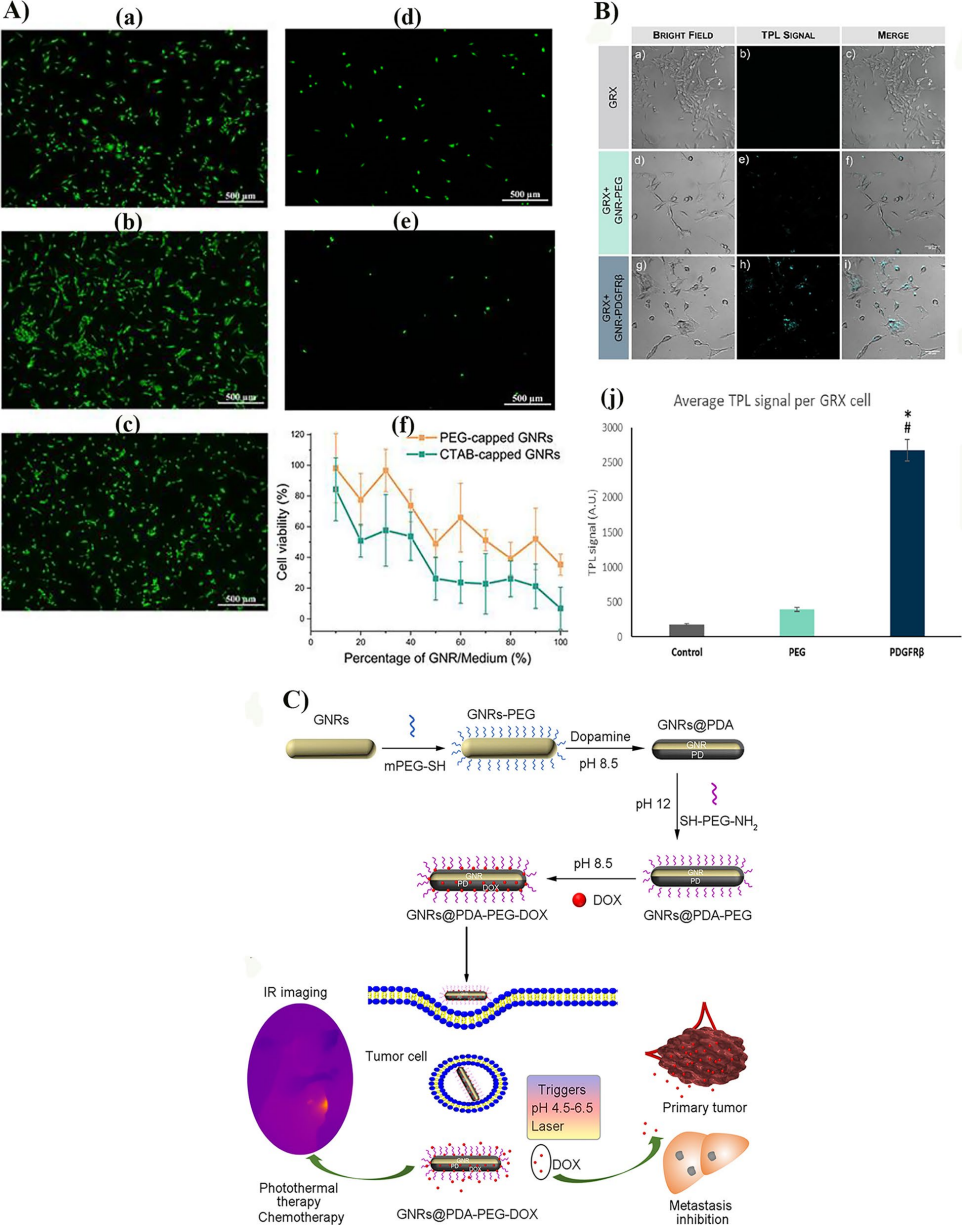
The interaction between functionalized AuNRs and cells, and the multifunctional AuNRs nanoplatform composite structure. **A** Cells that were incubated with (a) pure culture media, (b) 20% PEG-capped AuNRs, (c) 40% PEG-capped AuNRs, (d) 100% PEG-capped AuNRs, (e) 100% CTAB-capped AuNRs and stained with fluorescein diacetate (FDA), (f) cytotoxicity of AuNRs in HCC cell line. Reproduced with permission from Ref. [54]. Copyright 2019, Wiley Online Library. **B** (a) Specific uptake by immortalized normal primary mouse hepatic stellate cells (GRX) of AuNRs-PEG (panels d–f) and AuNRs-PDGFRβ (panels g–i) determined by TPL means. Bright-field and TPL merged images are shown demonstrating intracellular localization of AuNRs taken up. (b) Quantification of TPL signals from GRX cells incubated without AuNRs, with AuNRs-PEG and with AuNRs-PDGFRβ. Six fields of each condition were imaged and averaged. Data mean ± SEM.* indicates statistical differences compared to control group, and # compared to AuNRs-PEG, with *p* < 0.0001. Reproduced with permission from Ref. [60]. Copyright 2021, ACS Publication. **C** Synthesis procedure of GNRs@PDA-PEG-DOX nanocomposites and its applications for tumor ablation. Reproduced with permission from Ref. [66]. Copyright 2019, ACS Publication

The stabilizer, CTAB, is the leading cause of AuNRs toxicity; it is an important factor limiting the progress of AuNRs-PTT clinical trials. Replacing CTAB with biocompatible polymers such as PEG can reduce AuNRs toxicity in various cell types and murine models [52, 53]. PEG can remove approximately 88.9% of the CTAB bound to AuNRs surfaces, thereby improving biocompatibility and structural stability, preventing material morphology and thermal resistance changes, and changing AuNRs surface pharmacokinetics without affecting their photothermal ablation ability (Fig. 3A) [54, 55]. Polydopamine (PDA) promoted the functionalization of biomolecules on the surface of AuNRs, which could enhance the physiological stability and biocompatibility of the nanostructures, further allowing the AuNRs to achieve cell targeting and photothermal killing of cancer cells [56]. The core–shell composite obtained by coating polypyrrole (PPy) on the surface of AuNRs has good biocompatibility, photostability, and enhanced photothermal effect, showing high photothermal killing efficiency of cancer cells [57]. This composite structure can be used as a drug carrier for combination cancer therapy and opens up new ideas for cancer therapy, deep imaging, drug delivery, and diagnostic applications [57]. More and more biocompatible polymers are being developed to meet the requirement of reducing the biotoxicity of AuNRs while improving the protection of nanostructures. Appropriate polymer coating can also promote the further loading and transport of biomacromolecules such as antibodies or therapeutic drugs, and even enhance the optical penetration efficiency during PTT.

Further, the surface of AuNRs can bind specific antibodies, targeting peptides, phospholipids, and nucleic acids, such as DNA, small interfering RNA (siRNA), or small molecule drugs, which could improve the biological targeting of AuNRs [58, 59]. AuNRs-PDGFRβ formed by coupling AuNRs-PEG with anti-PDGFRβ antibody can precisely target the PDGFβ receptor on the surface of hepatic stellate cells, exerting an anti-hepatic fibrosis effect (Fig. 3B) [60]. The conjugation of AuNRs-PEG and cetuximab (CET) forms a novel smart nanoprobe (CET-PEG-AuNRs); in vitro and in vivo functional evaluation using NIR absorption imaging and PTT in related epithelial cancer has demonstrated its significant advantages in tumor diagnosis and treatment [61]. Double-peptide-modified AuNRs are formed by coupling EPPT-1 peptide targeting MUC-1 glycoprotein with myristoylated polyarginine peptide enhancing cellular uptake, so it has higher targeting and uptake efficiency in pancreatic ductal adenocarcinoma cells. Double-peptide-modified AuNRs can generate ionic nanobubbles under ultrashort-pulsed NIR laser light, causing local cellular environment disruption [62]. Furthermore, AuNRs can control DNA release in cells. For example, coupling PCR-amplified and thiol-modified EGFP-encoding DNA fragments with AuNRs transforms them into spherical particles that release DNA upon irradiation with femtosecond NIR pulsed laser light [63].

### Composite Structure-Based Multifunctional Nanoplatforms

Photothermal agents based on the AuNRs composite structure are the basis for realizing the nanophotothermal therapy multifunctional platform. Composite structure design mainly includes the combination of nanorods with related targeted therapeutic drugs, structural integration of nanorods and other nanomorphologies, surface material modification of nanorods, and optimization of the intrinsic structure of nanorods. Based on AuNRs nanophotothermal therapy, the role of the multifunctional nanoplatforms is to combine optoacoustic imaging, targeting functions of specific tissues and cells, and drug release to achieve precise diagnosis and treatment using nanomedicine.

AuNRs modified by magnetic Fe_3_O_4_ nanoparticles can synthesize a multifunctional nano-pearl necklace structure (AuNRs-Fe_3_O_4_); further, AuNRs-Fe_3_O_4_ can be functionalized by sulfhydryl-modified PEG and the antibody–drug Herceptin to construct multifunctional biological nanoprobes to target and thermally ablate human breast cancer cells (SK-BR-3) as magnetic resonance and fluorescence imaging agents [64]. The AuNRs@G-P-aspirin complex formed by the anti-inflammatory prodrug P-aspirin and AuNRs can inhibit PTT-induced inflammatory response by releasing aspirin during NIR light-triggered tumor thermal ablation [65]. AuNRs@PDA-PEG-DOX formed by coating AuNRs with doxorubicin (DOX) and polydopamine (PDA) is a multifunctional nanoplatform that integrates photothermal tumor ablation and tumor metastasis inhibition, carries tumor chemotherapy drugs to realize the combination of thermotherapy and chemotherapy, and completes PAI (Fig. 3C) [66].

The AuNRs core inside the mesoporous silica-coated AuNRs (AuNRs@SiO_2_) can be used as a two-photon imaging agent and thermotherapy agent; the mesoporous SiO_2_ shell has the potential for drug loading and can protect the internal AuNRs, which ensures that the chemotherapy provided by SiO_2_ shell and the hyperthermia provided by AuNRs core can function fully in a complex biological environment [67]. AuNRs@SiO_2_ nano-intraocular lenses with good biocompatibility and photothermal properties can precisely eliminate lens epithelial cells to prevent lens fibrosis and avert posterior capsule opacification after cataract surgery [68]. AuNRs can anchor and restrict the freedom of arginine-glycine-aspartic acid (RGD) sequences to bend flexible polypeptides into rigid conformations and use integrins to target tumor blood vessels to construct targeted multifunctional therapeutic agents with two-photon photoluminescence imaging and NIR photothermal conversion capability [69]. Through the electrostatic interaction, the highly sensitive microRNA-21 nucleic acid probe can be combined with AuNRs-PEI to detect the tumor-associated biomarker microRNA-21 in vivo with high sensitivity using fluorescence imaging and simultaneously enhance PAI and photothermal effects to diagnose and treat tumors effectively [70].

The AuNRs@MCs formed by microcube-encapsulated AuNRs can improve AuNRs targeting and reduce nanotoxicity; however, in contrast to the single AuNRs structure, its photothermal ablation and stability are not weakened because NIR light can easily pass through the microcubes and there are sufficient AuNRs to absorb NIR radiation to generate heat [71]. Cardiac cells can be implanted on nanocomposite scaffolds constructed from albumin electrospun fibers and AuNRs; this system, irradiated by 808 nm laser light, can induce AuNRs to convert light energy into thermal energy, increasing local temperature, changing the molecular structure of the fibrous scaffold, and attaching it firmly and securely to the heart wall, reducing the damage to tissues and organs caused using traditional suturing methods [72]. AuNRs can be self-assembled on DNA origami nanostructures to form D-AuNRs nanostructures, which can be used for PTT and PAI, and are crucial for enhancing imaging penetration depth, spatial resolution, and optical contrast in tumor diagnosis and treatment [73]. The hollow structure of hollow AuNRs with aspect ratios as low as 3 can be used for drug-loaded targeted delivery and to generate LSPR peaks in the NIR-II window to serve as NIR-II window-responsive small-sized nanoagents in PTT and PAI [74].

## AuNRs-PTT in Tumors

The photothermal conversion of AuNRs targeting tumor cells causes slow heat dissipation in structurally unsound tumor sites, and the resulting local hyperthermia of the tumor can alter tumor microcirculation. In addition, the thermal ablation can kill tumor cells and induce immune responses. Therefore, the effects of tumor AuNRs-PTT are based on the difference in biological effects between normal and diseased tissues and cells after absorbing thermal energy. AuNRs-PTT takes full advantage of the microscopic targeting positioning of nanorods and the tunability of NIR high photothermal conversion efficiency. These characteristics have attracted much attention in nanophotothermal therapy research.

### Improved Tumor-Targeting

Changing the size, shape, and surface conjugates of AuNRs can improve their tumor-targeting ability. Improving tumor-targeting and increasing the accumulation of AuNRs in the target tissue can reduce the damage to normal tissue caused by light irradiation and enhance the efficiency of PTT. Combining targeted AuNRs based on specific tumor markers with PTT can largely protect the surrounding normal cells and tissues. Medium-sized (70 nm × 11.5 nm) and surface-modified AuNRs with PEG and the tumor-targeting ligand lactoferrin (LF) (AuNRs@PEG-LF) exhibited the fastest cellular internalization in HepG2 cells, highest tumor aggregation in vivo, and the optimal in vitro photothermal effect [75]. The conjugation of AuNRs-PEG with anti-EGFR antibodies can be used for treating various epidermal growth factor-overexpressing cancer, including head and neck tumors, colorectal, ovarian, cervical, skin, breast, bladder, pancreatic, and prostate cancers [76].

Various tumor-targeting coatings based on natural and synthetic materials can improve AuNRs tumor-targeting. AuNRs modified with synthetic materials such as antibodies, peptides, and sugar molecules are easily internalized by tumor cells and cleared by the immune system. Natural materials exhibit better biocompatibility than synthetic materials; lipids and serum albumin are used for surface modification of nanostructures and help the modified nanostructures maintain colloidal stability and tissue targeting in the circulation system. Cancer cell membranes can also be used as a natural material, such as oral squamous cell membrane-coated AuNRs (AuNRs@Mem), which have good colloidal stability and tumor-targeting ability, and exhibit outstanding radiosensitization and cytotoxic capabilities induced by photothermal effect in vitro [77]. Protecting AuNRs with an enzyme-responsive zwitterionic stealth polypeptide coating in response to matrix metalloproteinase-9 overexpressed in the tumor microenvironment allows them to show a satisfactory systemic circulation half-life, significantly enhancing tumor cell uptake and markedly improving PTT in mouse models [78].

Tumor-targeting precision can be improved by designing drug delivery modes and vehicles. Loading AuNRs into existing targeted functional nanocarriers can ensure their stability and safety [79]. The study confirmed that interventional radiology to guide local vascular delivery of tumors, such as the delivery of AuNRs through the hepatic portal vein, could significantly increase the accumulation of nanoparticles in tumors and better target the liver tumor area than systemic delivery; CT-guided catheter interventional thermal ablation could locally damage tumor tissue while reducing damage to adjacent liver tissue [80]. Multifunctional microbubbles can synergistically deliver, control, and release AuNRs. AuNRs encapsulated in anti-vEGFR2-modified protein shell microbubbles (MBs) can target tumor site blood vessels. The MBs can be destroyed using ultrasound to release AuNRs, increasing transient cell permeability and enhancing AuNRs uptake by tumor cells, thus boosting the photothermal effect [81].

Cell-mediated nanoparticle drug delivery systems can reach many body areas normally inaccessible to common drugs or nanoparticles. BSA-coated macrophages loaded with 7-nm-diameter AuNRs utilize the inherent phagocytic ability of cells to optimize drug distribution within tumors, ultimately enhancing PTT efficiency [82, 83]. AuNRs@SiO_2_@CXCR4 nanoparticles were loaded into human-induced pluripotent stem cells (iPSCs) to construct a nanomaterial–cell system of AuNRs-iPSCs for PTT that exhibits strong migratory ability, targets gastric cancer sites, enhances PTT efficacy, and inhibits tumor growth in xenografted cancer cell mice at low laser power densities [84].

### Inhibition of Tumor Metastasis

**Fig. 4:**
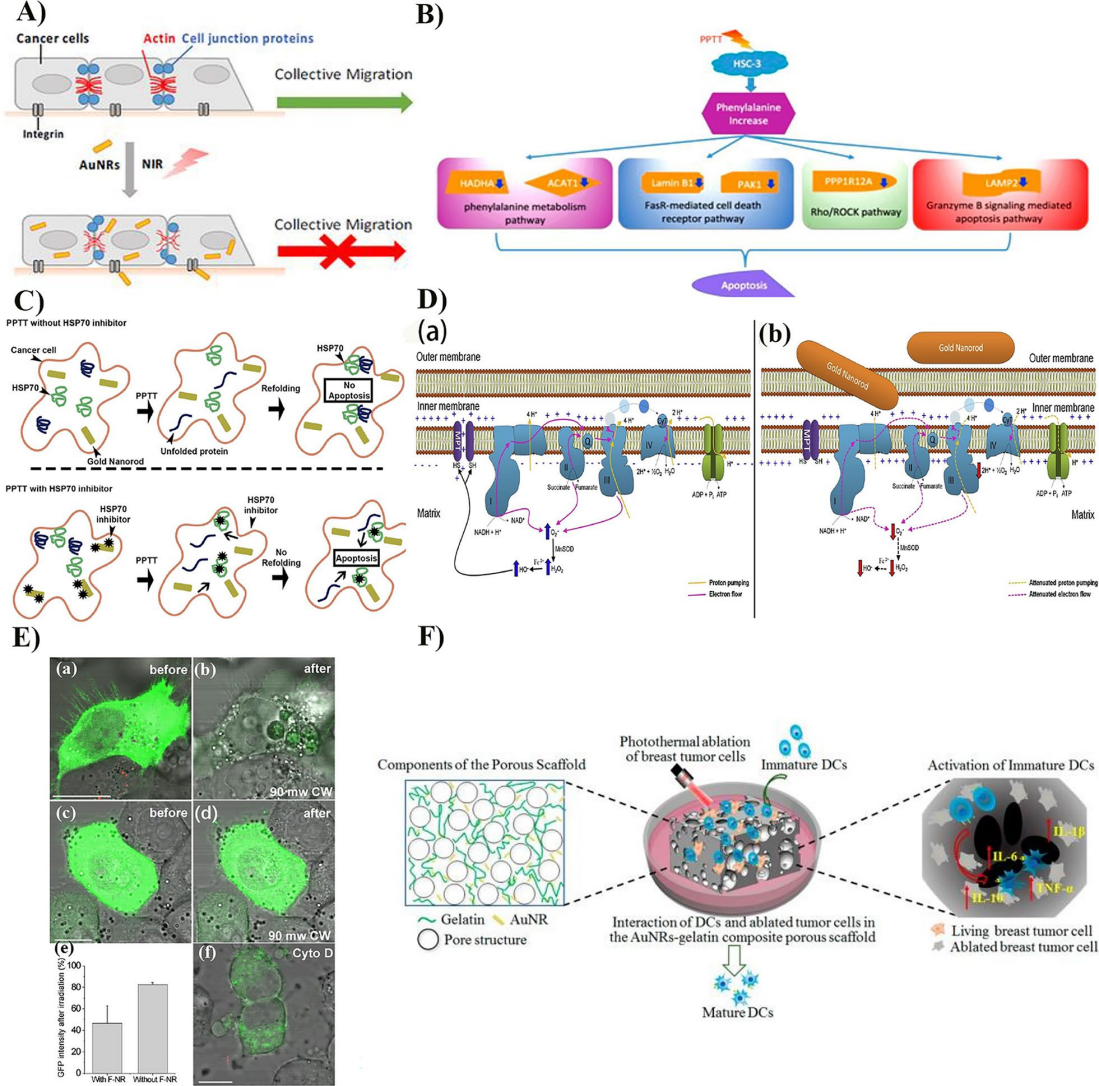
Mechanism of the photothermal tumor ablation and the tumor metastasis inhibition by AuNRs-PTT. **A** Targeting integrin could affect the actin cytoskeleton and cell junctions to result in the inhibition of cancer cell collective migration. Reproduced with permission from Ref. [85]. Copyright 2018, ACS Publication. **B** Schematic diagram explaining the molecular apoptosis mechanisms involved in altering phenylalanine metabolism as induced by PTT. Reproduced with permission from Ref. [90]. Copyright 2016, ACS Publication. **C** A model for HSP70 inhibitor optimized PTT. Reproduced with permission from Ref. [91]. Copyright 2016, Elsevier. **D** Mechanistic scheme. Mitochondrial dynamic scheme in the absence (a) and presence (b) of AuNRs. The four mitochondrial complexes are labeled as I, II, III, and IV. In the scheme, Mn-superoxide dismutase (MnSOD), glutathione peroxidase (GPx), peroxiredoxin (Prx), thioredoxin (TSH), glutathione (GSH), glutathione reductase (GR), thioredoxin reductase (TR), ubiquinone (Q), cytochrome c (CyT), and the mitochondrial permeability transition pore (MPT) are shown. Reproduced with permission from Ref. [95]. Copyright 2019, Elsevier. **E** F-AuNRs-mediated disruption of actin filaments in actin-GFP (green) transfected KB cells. (a) KB cell with internalized F-AuNRs (red) before cw laser irradiation. (b) Membrane blebbing accompanied by redistribution of actin-GFP and loss of fluorescence, after an 81.4-s exposure to cw irradiation at 90 mW. (c, d) KB cells without F-AuNRs, which did not experience membrane blebbing after exposure to cw irradiation at 90 mW. (e) Histogram showing the decrease in actin-GFP fluorescence intensity in cells with and without F-AuNRs labeling (*N* = 5) after cw irradiation. The minor reduction in fluorescence in cells without F-AuNRs labeling is attributed to photobleaching. (f) Blebbing, redistribution of actin-GFP, and loss of fluorescence in KB cells after 2-h treatment with cytochalasin D (5 μg/mL). Bar = 10 μm. Reproduced with permission from Ref. [93]. Copyright 2007, Wiley Online Library. **F** Illustration of the interaction of dendritic cells (DCs) and photothermally ablated tumor cells in gold nanorod–gelatin composite porous scaffolds. Reproduced with permission from Ref. [98]. Copyright 2019, MDPI (Basel, Switzerland)

Most cancer-related deaths are due to tumor metastasis. AuNRs-PTT can alter actin, cell junctions, and cellular energy metabolism, leading to cytoskeleton remodeling, thereby inhibiting tumor cell migration. Studies on signaling pathways, cytoskeleton, and cell connexin changes in AuNRs-PTT-stimulated cells confirmed that AuNRs-PTT could regulate and remodel actin filaments and cellular connexins and reduce the collective migration of cancer cells (Fig. 4A) [85]. RGD peptide-functionalized AuNRs can target overexpressed integrins in tumors that play a dominant role in cytoskeleton regulation, which disrupts focal adhesion, actomyosin contraction, and actin and microtubule assembly. Combined with PTT, they can lead to the reduction in lamellipodia and filopodia and the perturbation of integrin downstream regulatory molecules, further inducing cancer cell skeleton remodeling to inhibit cancer cell metastasis [86].

### Tumor Photothermal Ablation

Tumor photothermal ablation can treat digestive, respiratory, reproductive system, and lymph node tumors; human oral epidermoid and squamous cell carcinomas; and malignant melanoma [87–89]. The mechanisms of apoptosis, necrosis, and pyroptosis of tumor cell nanophotothermal ablation are worth exploring. Mass spectrometry-based metabolomics and proteomics combined with surface-enhanced Raman spectroscopy results during the implementation of PTT in tongue squamous carcinoma cells indicated that AuNRs-PTT significantly interfered with free phenylalanine and related metabolites to induce apoptosis (Fig. 4B) [90]. The viability of multiple cell lines was significantly decreased after HSP70 downregulation with siRNA and PTT treatment; simultaneously, apoptosis was increased (Fig. 4C) [91]. A molecular mechanism study results indicated that the apoptosis mechanisms of cytochromes c and p53 also enhanced AuNRs-PTT [92]. AuNRs can cause tumor cell death by impairing membrane integrity. When folate-coupled AuNRs (F-AuNRs) that could target folate receptors on the cell membrane surface were adsorbed on the cell membrane surface, the cell membrane was destroyed under NIR radiation, resulting in an influx of extracellular Ca^2+^, actin network degradation, and membrane vesicle production, leading to cell death (Fig. 4E) [93]. F-AuNRs can also inhibit the growth of human hepatoma cells (HepG2), associated with the cytoskeleton reorganization caused by cell membrane disruption and apoptosis due to Ca^2+^ influx [94]. The interaction of AuNRs with cellular mitochondria disrupts the electron transport chain, ultimately leading to cancer cell death (Fig. 4D) [95]. AuNRs-PEG can be internalized by cells and can enhance autofluorescence. In particular, AuNRs-PEG can double the autofluorescence of intracellular mitochondria that reflects the state of cellular respiration, allowing the autofluorescence of intracellular mitochondria to be monitored, tracking cell death during the treatment of intracellular AuNRs-PTT [96].

Photothermal ablation of tumor cells can trigger an immune response. After photothermal ablation of breast tumor cells with BSA-coated AuNRs, thermally ablated cells were cocultured with immature dendritic cells (DCs). The immune stimulatory responses of DCs could be induced by cell–cell interactions and soluble factors released by tumor cells [97]. On this basis, an AuNRs-gelatin composite porous scaffold with controllable pore size and good connectivity showed good photothermal efficiency and photothermal ablation ability for breast tumor cells. Photothermally ablated tumor cells cocultured with immature DCs on composite scaffolds induced DCs activation and triggered the immune system to prevent tumor metastasis and recurrence (Fig. 4F) [98].

### In vivo experiment of tumor PTT

In vivo experiments can be used to evaluate the circulatory kinetics of AuNRs and the safety and efficacy of AuNRs-PTT. AuNRs-PTT showed no pathologically significant toxicity to major organs in a mouse colon cancer model. The survival period of the photothermal treatment group was longer than that of the control group, and the AuNRs were quickly cleared from the blood and absorbed by the reticuloendothelial system [99]. The half-life of AuNRs in the circulatory system can be calculated by directly monitoring the surface plasmon bands of intravenously injected AuNRs in mice and measuring the pharmacokinetic parameters after intravenous injection of AuNRs in vivo. PC-AuNRs are easily recognized by the reticuloendothelial system due to their positively charged surface, and its half-life (1.3 min) is shorter than that of neutral-surface PEG-AuNRs (231 min) [100]. Given that AuNRs tend to be enriched in liver and spleen tissue, the subcellular localization of AuNRs in lysosomes was further demonstrated by selecting liver and spleen as target tissues, combining energy-dispersive X-ray spectroscopy of transmission electron microscopy. Synchrotron radiation X-ray absorption spectroscopy demonstrated that the long-term retention of AuNRs in the liver and spleen did not significantly affect the oxidation state of gold [101]. AuNRs-PTT treatment of mammary tumors in dogs and cats showed promising efficacy and was non-toxic to blood, liver, and kidney function [102]. However, the cost of in vivo experiments is high, and the procedure is uncertain. 3D bioprinted complex tissue can be used for quantitative photothermal characterization of AuNRs for early breast cancer treatment; it shows significant potential in elucidating the biological tissue variables of laser, AuNRs, and selective thermal damage [103].

### Photothermal Synergy with Various Therapies in Tumor Treatment

AuNRs are widely used in diagnostic and therapeutic research in which imaging and therapy complement each other. AuNRs-AlPcS4 (photosensitizer) complexes can be used in NIR fluorescence imaging of tumor sites to improve the therapeutic effect in vivo. Efficient PTT/PDT dual therapy is possible by combining AlPcS4 and AuNRs [104]. AuNRs labeled with folic acid and radioactive iodine can be used in SPECT/CT imaging to achieve thermal ablation of ovarian tumors overexpressing folic acid receptors and treat atherosclerosis, arthritis, and other diseases [105]. Single myeloma cells in the blood circulation can be detected using speckle modulation, optical coherence tomography, and AuNRs contrast agents to achieve dynamic detection and quantitative analysis of live circulating tumor cells [106].

PTT can synergistically enhance chemotherapy, radiation therapy, and heat-triggered drug delivery systems [107]. AuNRs combined with cisplatin killed 78% more tumor cells than cisplatin alone [108]. A study of plasmonic nanocarriers against cancer chemotherapy resistance has shown that AuNRs@SiO_2_-DOX inhibited the growth of drug-resistant breast cancer by suppressing the drug resistance pathway and increasing DOX accumulation. It can also reverse tumor cell drug resistance by increasing DOX accumulation and sensitivity through inhibition of drug resistance-associated genes [109]. The AuNRs–curcumin conjugate, AuNRs@Curcumin, releases curcumin by cleaving labile ester bonds in an esterase-rich tumor microenvironment, and photothermal treatment can accelerate curcumin release [110]. AuNRs@SiO_2_-DOX@CouC12-HA (GSDCH), constructed based on hydrophobic coumarin groups as blockers and smart control switches, can be efficiently taken up by human breast cancer cells. Subsequently, the coumarin layer is decomposed by NIR light to activate DOX release, realizing the synergistic effect of chemo-photothermal therapy [111].

PTT can be combined with gene therapy (gene silencing) to inhibit tumor growth and anti-tumor drug resistance. The combination of AuNRs-mediated mild heat therapy and oncolytic adenovirus gene therapy can effectively inhibit head and neck tumor growth [112]. The PEI-AuNRs/siRNA complex formed by siRNA adsorption on AuNRs functionalized on the surface of polyethyleneimine (PEI) can effectively deliver siRNA to breast cancer cells, and siRNA can resist pyruvate kinase M2 type (PKM2) to achieve gene silencing, significantly reducing the viability of breast cancer cells [113]. The siRNA of the glypican-3 (GPC-3) gene (*siGPC-3*), a new target for hepatocellular carcinoma (HCC) therapy, can induce specific gene silencing of GPC-3 in HCC, and galactose (GAL)-AuNRs-siGPC-3, obtained using GAL as the target portion of HCC, can induce GPC-3 gene silencing and photothermal effect to achieve tumor synergistic therapy [114]. In addition, the gene delivery system constructed by modifying AuNRs with low molecular weight polyethyleneimine exhibits good photothermal properties and gene delivery ability [115].

Photothermal immunotherapy also shows great potential. SiO_2_-AuNRs were loaded on human cytokine-induced killer (CIK) cells; in vitro and in vivo experiments showed that they targeted gastric cancer cells, enhanced gastric cancer tissue fluorescence and PAI, and improved immuno and photothermal therapies. As a “carrier,” CIK precisely carries functional nanoparticles or drugs to the tumor site to treat various tumors [116]. Amphiphilic poly-TLR7/8a and MMP-2-sensitive R9-PEG form AuNRs-IMQD-R9-PEG that effectively absorb tumor-derived protein antigens and directly from nanovaccines in vivo, enhancing the activation of host DCs, thereby amplifying adaptation anti-tumor T cell responses, triggering effector memory immune responses, and activating innate anti-tumor immunity [117]. The anti-tumor immune response involves a cascade of cancer immune cycles. A mild PTT based on AuNRs-doxorubicin gel, combined with antigen-trapping liposomes and anti-PD-L1 drugs, can promote the positive transfer of the cancer immune cycle [118].

Currently, PTT and PDT are the two most important phototherapy modalities, both of which have been accepted by the US Food and Drug Administration (FDA) as complementary technologies for the treatment of solid tumors [119]. PTT utilizes light-absorbing substances to generate heat from light, resulting in thermal ablation and subsequent cell death of cancer cells [13]. PDT involves the administration of a photosensitizer followed by irradiation with light of the appropriate wavelength to induce cell death, and the entire reaction produces reactive oxygen species (such as singlet oxygen and free radicals), which are toxic and capable of killing cancer cells [13]. Compared with PDT, PTT has less chemical toxicity and focuses more on the use of physical processes to achieve therapeutic purposes, trying to achieve hyperthermia treatment or thermal ablation of cancerous sites through a non-toxic and noninvasive process. However, in the process of clinical application, PTT is far less successful than PDT. The main reason is that the controllability of the photothermal process is relatively poor, and the positioning and role of heat in the living structure have not been systematically grasped. Therefore, the synergistic treatment of PTT and PDT still needs more attention. The multifunctional anionic photosensitizer indocyanine green-conjugated AuNRs can act as both photodynamic and photothermal agents to destroy cancer cells [120]. Combination therapy of PDT and PTT can kill cancer cells more effectively, and the system can also serve as an effective bioimaging probe in the near-infrared region [120].

## AuNRs Photothermal Treatment of Inflammatory Diseases; Killing Bacteria and Viruses

**Fig. 5:**
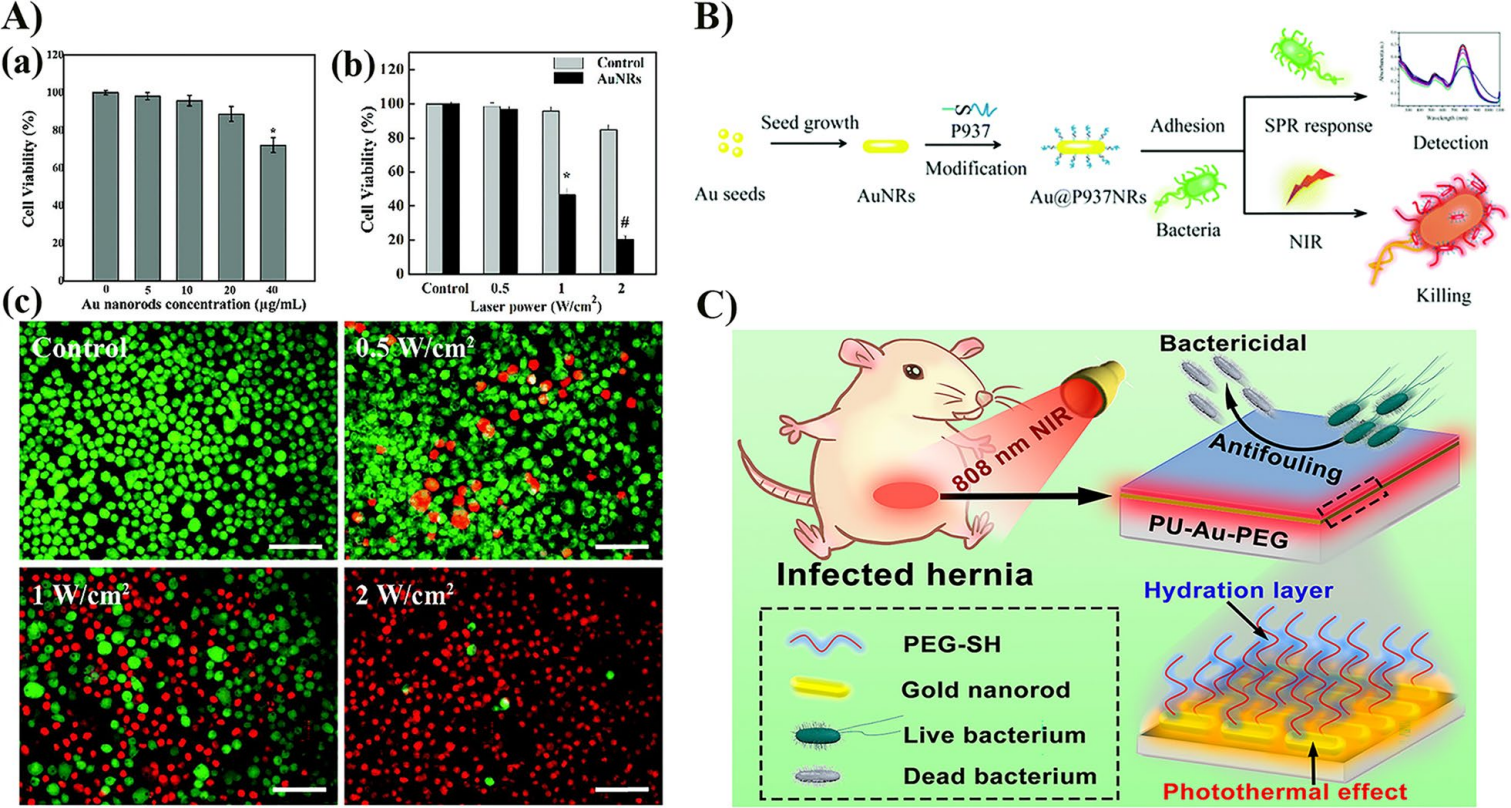
AuNRs photothermal treatment of inflammatory diseases; killing bacteria and viruses. **A** Cytotoxicity of AuNRs and PTT. (a) In vitro cell viability of Ana-1 cells, a mouse macrophage cell line, treated with or without various concentrations of the AuNRs for 24 h. (b) Cell viability of Ana-1 cells treated with or without the AuNRs (20 μg mL^−1^) after the NIR laser irradiation with different power densities (0, 0.5, 1, and 2 W cm^−2^). (c) Live cells and dead cells were stained with calcein AM (green) and PI (red), after incubation of Ana-1 cells with or without the AuNRs (20 μg mL^−1^) and being exposed to an 808 nm laser at different power densities (0.5, 1, and 2 W cm^−2^). Scale bar: 100 μm. Reproduced with permission from Ref. [121]. Copyright 2015, Royal Society of Chemistry. **B** Au@P937 NRs for detecting bacteria by specific bacterial binding and killing bacteria due to the local hyperthermal effect. Reproduced with permission from Ref. [123]. Copyright 2018, Royal Society of Chemistry. **C** Schematic Illustration of NIR-Responsive PU-Au-PEG Surface with Antifouling and Photothermal Bactericidal Properties. Reproduced with permission from Ref. [125]. Copyright 2020, ACS Publication

Even at relatively low AuNRs concentrations and low NIR power, AuNRs-PTT can exhibit a significant macrophage-killing effect. Relevant in vivo experiments demonstrate that AuNRs are effective in in vivo imaging and PTT of inflammatory macrophages in femoral artery restenosis; inflammatory macrophages also play a key role in developing atherosclerosis (Fig. 5A) [121]. A nanoscale drug delivery system using AuNRs as a photothermal agent and mesoporous silica shell as the methotrexate storage body exhibits good photothermal conversion efficiency with specifically targeted cytotoxicity against activated macrophages under NIR laser irradiation. Local irradiation-induced hyperthermia ensures that AuNRs-PTT can be used to treat rheumatoid arthritis with chemotherapy synergistically [122].

AuNRs-PTT can be used to detect and kill microbial strains. The longitudinal LSPR absorption peak of bifunctional Au@P937 nanorods obtained by coupling adhesive peptides with AuNRs is sensitive to changes in bacterial concentration; the positively charged surface of Au@P937 nanorods and the affinity of peptide P937 facilitate the binding of nanorods to the negatively charged bacterial surface; therefore, pathogenic bacteria can be detected based on variations in the LSPR band. In addition, pathogenic bacteria can be quickly killed by 808 nm laser irradiation (Fig. 5B) [123]. AuNRs-PEG and polystyrene hydrophobic-functionalized (PS-AuNRs) enhance the antibacterial activity of AuNRs at PTT and effectively kill *Staphylococcus aureus* and *Propionibacterium acnes* [124]. The photothermal killing of bacteria by AuNRs can be applied to biomedical device-associated infections. The preparation of AuNRs-PEG coating on the surface of biomedical polyurethane (PU) can give it natural antifouling and photothermal sterilization properties; its in vivo photothermal antibacterial effects have been confirmed in animal experiments and clinical settings (Fig. 5C) [125]. An aptamer conjugated RVG-Apt-PEG-SiO_2_-AuNRs-mediated PTT was applied to neurotropic rabies virus (RABV) infection. Nanostructures obtained by coupling DNA aptamers and rabies virus glycopeptides to AuNRs can specifically target RABV glycoproteins in cells and the mouse brain to enhance the central nervous system delivery function. Viral RNA levels and RABV distribution in the mouse brain are significantly reduced after PTT administration, providing a promising strategy for the clinical treatment of neurotropic viral infections [126].

PTT can regulate the microbial environment around the wound and effectively prevent wound infection to promote wound healing. However, the high temperature in PTT treatment not only kills bacteria, but also causes damage to surrounding normal tissues and cells. In addition, the bacterial targeting of PTT and the stability and durability of wound treatment are still not high. Controlled sterilization using mild temperatures allows wound healing without thermal damage. A novel antibacterial nanosystem based on AuNRs, Dap@Au/Ag nanorods, can release a large amount of silver ions and antibacterial peptides (Dap) under the action of mild photothermal heating and hydrogen peroxide, which destroys the integrity of bacterial membranes and leads to death of bacteria [127]. The mild PTT achieved by this method effectively reduced the incidence of skin burns and successfully avoided secondary infections caused by burns.

## Optimization of Nanomaterials and Improvements in NIR Irradiation Conditions

One of the core issues of AuNRs-PTT is how to improve and control the interaction of biological structures, nanomaterials, and NIR irradiation. Biological structures, including cells, tissues, and organs, are PTT targets with significant individual differences. The optimization of AuNRs and the improvement in NIR irradiation are the basis of in vivo, in vitro, and preclinical experiments. As a novel photothermal agent, the intrinsic rod structure, in vitro cytotoxicity, in vivo tissue distribution, tissue and cell targeting, and organ delivery mechanisms of AuNRs directly affect the success or failure of PTT [128, 129]. The NIR irradiation conditions are essentially an external field applied to nanomaterials and biological structures, and the intensity, duration, area, and form of irradiation are directly related to the efficiency of photothermal conversion, inflammatory response, and damage to normal cells and tissues caused by irradiation with AuNRs-PTT [130, 131].

At suitable particle sizes, AuNRs can provide optimal NIR light absorption and scattering and are more easily absorbed by cells [132]. For large nanorods (e.g., longitudinal and transverse lengths of 38 nm × 11 nm), strong light scattering decreases absorbance, whereas for small nanorods (e.g., longitudinal and transverse lengths of 17 nm × 5 nm), it is not sufficient to allow coupling between the electric fields of adjacent nanorods in solution, reducing the photothermal conversion; therefore, only AuNRs with suitable longitudinal and transverse lengths (e.g., 28 nm × 8 nm) are optimally effective in AuNRs-PTT [133]. The uniform temperature increase in the AuNRs target tissue is important in the thermal ablation process of PTT, and the concentration of AuNRs is a key factor for uniform temperature increase in the target tissue area [134]. It is also necessary to pay attention to the LSPR peak position of AuNRs. AuNRs with LSPR in the first NIR window (NIR-I, ~ 800-nm longitudinal LSPR peak) exhibit optimal photothermal treatment effects [135]. However, the gold nanoshell rod structure obtained by encapsulating AuNRs cores in AuAg nanoshell cavities can photothermally ablate tumor cells in the first NIR (NIR-I) and second NIR (NIR-II) windows [136]. Gold islands and gold nanowires can be grown on AuNRs; adjusting the degree of gold nanowires branching can form Au-on-AuNRs hybrid structures with tunable LSPR peaks in the entire visible-NIR region that exhibit strong absorption and excellent photothermal conversion in the NIR-II region [137].

Pulsed laser light has a significant effect on the external structure of AuNRs. Light-induced local energy deposition and dynamic stress distribution can cause changes in the atomic structure that affect the shape and related properties of AuNRs, leading to variations in their photothermal properties in practical applications [138]. Low-energy NIR light (700 − 1700 nm) with low phototoxicity and high tissue penetration depth is the preferred irradiation condition for AuNRs-PTT [139, 140]. Analysis of the thermal effects on rat breast cancer tumor models using different combinations of NIR wavelengths and AuNRs showed that 808 nm and 1064 nm laser light produced the greatest AuNRs temperature enhancement. Since 808 nm laser light has the fastest laser-induced thermal response rate, it is most widely used in AuNRs-PTT [141]. Most of the laser light exposure required for effective PTT significantly exceeds the maximum allowable power density for human skin and can potentially damage the surrounding normal tissue. LED light sources can be safer than laser light, and when acting in concert with AuNRs to produce photothermal effects, they can quickly and efficiently heat cells and tissues to ablation temperatures [142]. Radially polarized beams can excite randomly oriented AuNRs, reducing the energy required to kill cancer cells, with less damage to surrounding tissue. The energy flux required for AuNRs-PTT of relevant cancer cells can be reduced to one-fifth of that required using linearly polarized light [143]. Temperature monitoring, laser irradiation conditions (power and spot radius), and tumor aspect ratios closely relate to the tumor apoptosis rate during the hyperthermia process. Damage to normal tissues around the photothermal treatment area is avoided as much as possible only by determining the optimal tumor aspect ratio and laser irradiation conditions for PTT [144, 145].

## Conclusion and Outlook

In summary, this review systematically describes the application of AuNRs as nanophotothermal therapeutic agents in tumor hyperthermia and thermal ablation, inflammatory diseases, bacteria- and virus-killing; it focuses on functionalized AuNRs and the interaction between nanostructures and cells and shows that the interaction between biological structure, nanomaterial, and NIR irradiation is the essence and basis of AuNRs-PTT. As previously mentioned, optimizing the biocompatibility and targeting of AuNRs and constructing multifunctional nanoplatforms with composite structures could enhance the performance of nanophotothermal agents. It could also improve NIR irradiation conditions to accommodate the nanophotothermal effect, which is the key to improving AuNRs-PTT. Studies are increasingly being conducted to develop less toxic AuNRs through surface modification and validate the therapeutic effects of nanophotothermal therapy on various diseases through in vitro cellular experiments and in vivo animal models while keeping the normal organism intact. AuNRs-PTT could occupy an important position in emerging noninvasive nanomedicine treatments; however, many challenges must be faced to achieve this goal. First, the numerous functionalized forms of AuNRs structures and compositions add to the complexity of their study. As a plasmonic inorganic nanomaterial, the intrinsic cytotoxicity introduced during the preparation of AuNRs can be complicated by multiple functionalized modifications, which are related to the transport, distribution, excretion, degradation, and safety of nanostructures in living systems. Reducing the complexity of AuNRs preparation and improving the evaluation mechanism of nanomaterial cytotoxicity are the basis on which AuNRs-PTT could be sufficiently perfected to move toward clinical research and application. Second, it is worth exploring the coordination of biological structures, nanomaterials, and NIR irradiation to achieve the optimal AuNRs configuration. Various cells, tissues, and organs respond differently to nanostructures and external field irradiation; therefore, effective functionalized AuNRs and NIR conditions for treating one tumor may not be effective for another. Understanding the response law of biological structures to nanostructures and external irradiation, and then predicting and designing the conditions of nanophotothermal agents and optical irradiation are crucial for mature clinical research and the application of AuNRs-PTT. In conclusion, although the current AuNRs-PTT is still far from clinical translation, we believe its potential in clinical diagnosis and treatment, cancer cure, drug development, and precision medicine is significant.

## Data Availability

Not applicable.

## Abbreviations

AgNO3: Silver nitrate
AuNRs: Gold nanorods
BSA: Bovine serum albumin
CET: Cetuximab
CIK: Cytokine-induced killer
CTAB: Cetyltrimethylammonium bromide
DCs: Dendritic cells
DOX: Doxorubicin
EPR effect: Enhanced permeability and retention effect
GAL: Galactose
HCC: Hepatocellular carcinoma
HCL: Hydrogen chloride
iPSCs: Induced pluripotent stem cells
LF: Lactoferrin
LSPR: Localized surface plasmon resonance
MBs: Microbubbles
NIR: Near-infrared
PAI: Photoacoustic imaging
PC: Phosphatidylcholine
PDA: Polydopamine
PDT: Photodynamic therapy
PEG: Polyethylene glycol
PEI: Polyethyleneimine
PKM2: Pyruvate kinase M2
PPy: Polypyrrole
PTT: Photothermal therapy
PU: Polyurethane
RABV: Rabies virus
RGD: Arginine-glycine-aspartic acid
siRNA: Small interfering RNA
TPI: Two-photon imaging
UV–Vis: Ultraviolet–visible spectroscopy
